# Are the current feeding volumes adequate for the growth of very preterm neonates?

**DOI:** 10.1017/S0007114523000338

**Published:** 2023-10-28

**Authors:** Chrysoula Kosmeri, Vasileios Giapros, Antonios Gounaris, Rozeta Sokou, Ekaterini Siomou, Dimitrios Rallis, Alexandros Makis, Maria Baltogianni

**Affiliations:** 1 Department of Pediatrics, University Hospital of Ioannina, Ioannina, Greece; 2 Neonatal Intensive Care Unit, School of Medicine, University of Ioannina, Ioannina, Greece; 3 Neonatal Intensive Care Unit, School of Medicine, University of Larissa, Larissa, Greece; 4 Neonatal Intensive Care Unit, Nikaia General Hospital ‘Aghios Panteleimon’, Athens, Greece

**Keywords:** Very preterm neonate, Postnatal growth failure, Nutrition, Feeding volume

## Abstract

Postnatal growth failure, a common problem in very preterm neonates associated with adverse neurodevelopmental outcome, has recently been shown not to be inevitable. There is a wide discussion regarding feeding practices of very preterm neonates, specifically regarding feeding volumes and nutrients supply to avoid postnatal growth failure. Current guidelines recommend an energy intake of 115–140 kcal /kg per d with a considerably higher upper limit of 160 kcal/kg per d. The feeding volume corresponding to this energy supply is not higher than 200 ml/kg in most cases. From the other side, randomised and observational studies used higher feeding volumes, and these were associated with better weight gain and growth, while no complications were noted. Taking into account the above, nutritional practices should be individualised in each very and extremely preterm infant trying to reduce postnatal growth failure, pointing out that available data are inconclusive regarding the effect of high-volume feeds on growth. Large clinical trials are necessary to conclude in the best feeding practices of very preterm neonates.

## Graphical abstract

**Figure:**
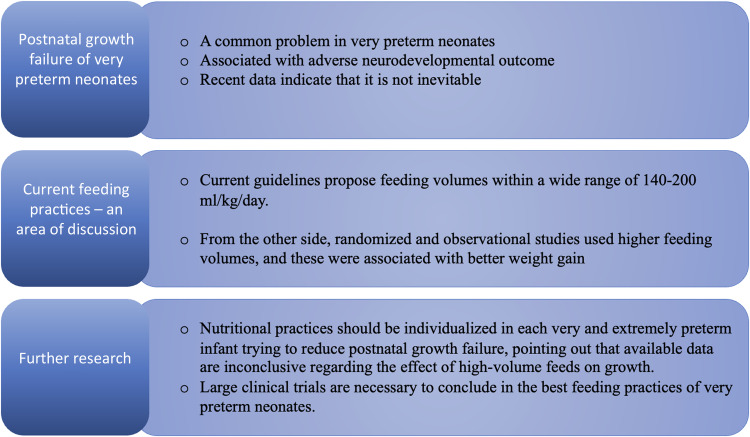

Despite advances in neonatal care and nutritional practices, postnatal growth failure remains common among very preterm neonates^(1,2)^. In a multicentre study^(3)^ of 1187 infants born at 23–27 weeks of gestation at fourteen neonatal intensive care units in the USA, postnatal growth failure was found in 75 % of these infants at 28 d of life even though the growth velocity rate was above 15 g/kg per d, which is considered an adequate growth velocity for preterm infants^(4–6)^. For many years, the goals of nutritional care are set to approximate the growth and body composition of a healthy fetus although it is recognised that optimal proportions of fat and lean mass accretion will differ^(7,8)^.

Postnatal growth failure is of great concern since there is evidence that it is associated with poor neurodevelopmental outcomes^(4,9)^. A multicentre cohort study that evaluated 495 infants with 501 to 1000 g birth weight found that the growth velocity had a significant and possibly independent effect on neurodevelopmental and growth outcomes at 18–22 months corrected age^(4)^. Similarly, a study of 219 very low birth weight infants showed that children with postnatal weight gain below the 10th percentile at the age of 2 years were at the highest risk for mental retardation, motor delay, and cerebral palsy, and their developmental outcome was even worse than that of children who were small for gestational age and had insufficient catch-up growth. Therefore, this study concluded that the postnatal growth pattern was the most important factor associated with adverse neurodevelopmental outcomes at the age of 2 years^(9)^. However, the association of postnatal weight gain and head growth with later neurocognitive outcomes was reported mainly in observational studies but not in interventional studies^(10)^.

For the last 20 years, there is a discussion regarding the prevention of postnatal growth failure especially in very preterm infants with major morbidities^(11)^. Population-based studies initially proposed that postnatal growth failure was inevitable^(12,13)^. In 2018, Andrews et al analysed data from 396 preterm very low birth weight newborns in a tertiary neonatal unit after the implementation of new nutritional practices. They found that most infants had growth approximating their birth centile, indicating that adequate postnatal growth was not inevitable^(14)^. Several reasons avert very preterm neonates to achieve optimal growth. Embleton et al in 2001 stated in their review that preterm infants had a significant and irreplaceable nutrient deficit in the first few weeks of life that led to postnatal growth failure. This deficit was due to nutritional practices that were based on nutrient maintenance and normal growth but not on catch-up growth^(15)^. The problem seems to be more severe in very preterm neonates with co-morbidities such as bronchopulmonary dysplasia (BPD). Very preterm neonates with morbidities had further reduction in growth during hospitalisation,^(16)^ and this might be due to lower energy administration than that recommended by current feeding practices leading to a further increase in energy deficit^(11,17)^. The observations above indicate that a energy deficit is accumulated, which should be taken into consideration in nutritional practices during hospitalisation. This practice is also emphasised in a recent statement by ESPGHAN who recommended increasing nutrient intakes above estimated target needs during the recovery phase^(18)^.

## Target feeding volumes and energy supply

Strategies for the prevention of malnutrition and the resulting insufficient growth have been studied including the volume of the feeds. The range of feeding volumes that is discussed in the widely implemented clinical practice 2010 ESPGHAN guidelines lies within 150–180 ml/kg per d (aiming to 110–135 kcal/kg per d energy) with lower and upper limits of 135–200 ml/kg per /d^(19)^. Studies from 127 tertiary neonatal intensive care units reported feeding a wide range of feeding volumes of 140–200 ml/kg per d^(20)^. The very recent update of ESPGHAN guidelines^(8)^ recommends a higher energy supply (115–140 kcal/kg per d *v*.110–135 kcal/kg per d the older guidelines) with a considerably higher upper limit of 160 kcal/kg per d. The feeding volume corresponding to this energy supply is not higher than 200 ml/kg in most cases. Moreover, in the same report, emphasis is given to balance among the several nutrients (energy fractions) for optimal nutrition to promote optimal growth and long-term outcomes^(8)^. Of note, these proposed volumes refer to fortified human milk, which is the best practice or alternatively special formula for very preterm neonates. All but one studies discussed below refer to respective volumes of the above-mentioned type of feeding.

As ESPGHAN Committee on Nutrition and invited experts in a recent position paper emphasise, these recommendations do not consider changes in energy needs related to acute illness or chronic disease states^(8)^. These conditions are very common in this population. They do not also consider additional nutrient losses or demands of very preterm neonates^(8)^. They also conclude that in individual preterm infants, enteral intakes up to 200 ml/kg per d (or higher) may be appropriate and safe depending on current health status, such as the presence of a significant patent ductus arteriosus or BPD^(8)^. They also discuss the relevance in specific contexts of the administration of volumes above 200 ml/kg per d, namely in low-middle income countries where access to human milk fortifiers is limited or in infants that do not tolerate full-strength fortification.

In a randomised clinical trial of 224 infants born very preterm weighing 1001–2500 g at birth, volume feedings of 180–200 ml/kg per d of fortified human milk increased growth velocity, weight, head circumference, length and mid-arm circumference compared with usual-volume feedings of 140–160 ml/kg per d of fortified human milk^(21)^. The average growth velocity of infants in the higher-volume group was 20 g/kg per d,^(21)^ higher than the 15 g/kg per d that is considered adequate for catch-up growth^(5,6)^. Andrews et al analysed data from 396 preterm neonates and found that optimal nutritional practices led to infants’ growth approximating their birth centiles^(14)^. Another smaller randomised study of sixty-four preterm infants with birth weight < 1500 g found that infants fed on a high volume of expressed breast milk (300 ml/kg per d) had significantly higher daily weight gain compared with infants fed on a maximum of 200 ml/kg per d^(22)^. However, this study did not provide any data on the length and head circumference increase between the two groups^(22)^. Similarly, in a trial of fifty-four infants born at 24–29 weeks of gestation, volume feedings of 200 ml/kg per d of fortified human milk or preterm formula increased growth velocity, weight gain and arm fat area of infants, compared with those fed at volumes of 150 ml/kg per d^(23)^. However, head circumference and length did not differ significantly^(23)^. An older study of fifty-nine preterm infants born 1–2 kg birth weight also reported weight gain at the intra-uterine rate with feeding volumes of 250 ml/kg per d of breast milk or standard formula^(24)^. The available data indicate that higher feeding volumes led to higher weight gain and optimal growth velocity for catch-up growth. A recent Cochrane review of two randomised controlled trials comparing high *v*. standard enteral feeds for preterm or low birth weight infants concluded that high-volume feeds probably improve weight gain during the hospital stay, yet available data are inconclusive on the effect of high-volume feeds on growth and clinical outcome^(25)^.

On the other hand, fear of high-volume-related complications, mainly patent ductus arteriosus and BPD^(19)^, leads to hesitation in the advancement of feeding volumes. Available studies find no adverse effects in the higher volume groups regarding fluid retention, haemodynamically significant patent ductus arteriosus, tachypnoea, rate of BPD, duration of respiratory support, necrotising enterocolitis, feeding intolerance and length of stay^(21–24,26)^. In a recent study of very preterm infants, gradually advancing milk feedings up to 260 ml/kg per d were generally well tolerated and without side effects^(26)^. Neonates with BPD did not have a significant difference in their respiratory function at 8 years of age compared with very preterm neonates without BPD and term controls when fed with increased milk volumes^(27)^.

Another possible clinicians’ concern is that higher feeding volumes may cause emesis or reflux or even just worsen reflux with a subsequent impact on respiratory status. Current studies assessing higher feeding volumes evaluated preterm infants for signs of feeding intolerance, such as episodes of vomiting and increased aspirates. In the study of Thomas et al, more infants in the high-volume group had feeding intolerance; however, this observation was not statistically significant^(22)^. No difference in feeding intolerance between higher and standard feeding volumes was also noted in two other studies^(23,28)^. A meta-analysis also found that there is little or no difference in feeding intolerance between shorter feeding intervals (smaller milk volumes) and longer intervals (higher milk volumes) in very preterm infants^(29)^. A recent randomised controlled trial of 2804 very preterm or very-low-birth-weight infants found that daily milk increments of 30 ml/kg *v*. 18 ml/kg did not make a difference in survival without moderate or severe neurodevelopmental disability, late-onset sepsis or necrotising enterocolitis^(30)^. A Cochrane review of six trials concluded that data are insufficient to determine how progressive introduction of enteral feeds affects the risk of necrotising enterocolitis (NEC)^(31)^. Another recent Cochrane review of fourteen trials involving 4033 infants showed that slow advancement of enteral feed volumes probably has little or no effect on the risk of NEC and overall-cause mortality^(32)^. In this review, meta-analyses suggested that slow advancement may slightly increase feed intolerance and the risk of invasive infection; however, this evidence was of low certainty^(32)^.

Another study using a similar intensive feeding policy did not find to affect the BMI and obesity rates at the ages of 2 and 8 years^(26)^. Moreover, rapid weight gain up to term-corrected age in preterm infants had no impact on later metabolic status, while rapid weight gain in later childhood seemed to affect the cardiometabolic status^(33)^. Body composition is not routinely estimated in neonatal units, whereas all three anthropometrics namely head circumference, body weight and body length, as a proxy for lean mass accretion, should be taken into consideration. Optimally, growth in the preterm neonates should not lead to excessive fat deposition, and this is addressed in the last ESPGHAN guidelines also^(8)^. Low energy intake in the first week of life may be a predisposing factor for complications. A large Swedish retrospective study of 498 infants less than 28 weeks gestation showed that a low energy intake of 102 kcal/kg per d during the first 4 weeks of life was an independent risk factor for the development of severe retinopathy of prematurity, while an increase in energy intake of 10 kcal/kg per d was associated with a 24 % decrease in severe retinopathy of prematurity^(34)^.

## Discussion and conclusion

Very preterm neonates vary in many aspects such as gestational age, birth weight, sex, perinatal complications, type of feeding (fortified human milk which is the best practice or alternatively special formula or combination), heritability (parental BMI and obesity) and epigenetic factors. Studies in young adults (although reluctance exists when extrapolating adult data to neonates) emphasised the crucial role of heritability and other factors such as gut microbiota in weight gain^(35,36)^. Based on the above parameters, it seems unlikely that a standard amount of energy fits the needs of each preterm infant to achieve a standard growth pattern and that the amount of feeding should be individualised. Factors such as appetite and satiety regulation which seem to play a crucial role in delineating the true physiological needs to maintain a healthy, normal weight have not so far been studied in preterm neonates^(37)^. There are also no studies assessing the association of nutrition and constitutional factors in the growth of preterm infants. An early study of Kuschel et al demonstrated that almost half of the infants required higher milk intakes to maintain weight gain and the other half required a reduction of milk intake due to feed intolerance. This observation further supports the need for individualisation of feeding^(23)^. In corroboration of our notions in the recent ESPGHAN statement, the authors highlight that growth in the *ex utero* environment will never be the same as *in utero*, that optimal proportions of fat and lean mass accretion will differ, and that optimal nutrient intakes and growth trajectory for an individual infant are impossible to determine^(8)^.

Currently available guidelines propose feeding the preterm newborn to provide an energy supply of 115–140 kcal/kg per d (maximum 160 kcal/kg per d) which typically represents a feeding volume of 140–180 ml/kg per d (maximum 200 ml/kg per d)^(8)^. In the previous and largely adopted 2010 ESPGHAN statement, whereas slightly smaller volumes have been proposed, the authors state that these feeding volumes may be inadequate for several substrates and have accepted that the nutrient intake above this specified range is not discouraged if justified for a good reason^(19)^. Similar concerns are expressed in the current report also, whereas authors acknowledge that neonatal nutrition research is extremely active, and it is likely that alternative approaches and recommendations may be preferable as our knowledge expands. Special preterm formula has a standard concentration with increased protein content; thus, in case of inadequate weight gain, the quantity should be increased since the energy and protein ratio needs to be stable, a necessary issue for protein utilisation. We have observed that very preterm neonates often need higher feeding volumes of fortified human milk or special preterm formula or combination to maintain minimum weight gain. Moreover, we have observed in three tertiary neonatal care units (unpublished observations) that many very preterm infants, immediately after the transition from tube feeding to bottle feeding, consume *ad libitum* milk volumes of 250–300 ml/kg per d or even higher. Recent studies conducted by our research group showed that these higher feeding volumes are usually well tolerated and have not adversely affected BMI at school age, and moreover, very preterm neonates treated with a more intense feeding policy had a good respiratory prognosis at school age^(26,27)^. Furthermore, healthy bottle-fed full-term neonates and young infants followed up in our outpatient clinic usually consume variable milk volumes ranging between 150 and 250 ml/kg, but their weight gain is also variable and, on several occasions, unrelated to the consumed milk volumes. These observations have also been made by other researchers^(22,24,38,39)^. In a few studies, volumes up to 300 ml/kg per d have been administered earlier but also recently^(22,24,26,38,39)^. One could speculate that reluctance in milk administration exists among many neonatologists in tube-fed premature babies and that many physicians rely on unproven risks to prescribe milk with caution. The interplay between growth factors, genes, epigenetics and nutrient supply, on an individual basis, may affect the early postnatal growth in the preterm infant^(40)^. Nutrient supply is the parameter that we can easily modulate to achieve optimal short-term and long-term outcomes.

In conclusion, nutritional practices should be individualised in each very preterm infant for ideal growth. Available data are inconclusive regarding the effect of high-volume feeds on growth and later outcomes. There is a need for larger randomised studies that compare higher target feeding volumes in very preterm infants to universally conclude in the optimal feeding practices.
